# Higher serum myostatin levels are associated with lower insulin sensitivity in adults with overweight/obesity

**DOI:** 10.14814/phy2.16169

**Published:** 2024-09-11

**Authors:** Laura E. Dichtel, Allison Kimball, Bryan Bollinger, Geetanjali Scarff, Anu V. Gerweck, Miriam A. Bredella, Melanie S. Haines

**Affiliations:** ^1^ Neuroendocrine Unit, Department of Medicine Massachusetts General Hospital Boston Massachusetts USA; ^2^ Harvard Medical School Boston Massachusetts USA; ^3^ Department of Radiology Massachusetts General Hospital Boston Massachusetts USA

**Keywords:** insulin sensitivity, muscle, myostatin, obesity

## Abstract

Myostatin inhibition improves insulin sensitivity in preclinical and clinical models; however, studies investigating the relationship between serum myostatin levels and insulin sensitivity are discrepant. Sensitive and specific myostatin LC–MS/MS assays are now available to accurately assess serum myostatin level in vivo. We sought to determine whether higher serum myostatin levels are independently associated with lower insulin sensitivity in adults with overweight/obesity. Participants included 74 adults, 20–65 years old, BMI *≥*25 kg/m^2^ without type 2 diabetes. Appendicular lean mass (ALM) was measured by dual‐energy x‐ray absorptiometry; visceral adipose tissue (VAT) was measured by computed tomography. Main outcome measures were serum myostatin levels (LC–MS/MS) and insulin sensitivity (Matsuda index). Mean age was 48 ± 12 years, and BMI was 33.1 ± 5.6 kg/m^2^ (mean ± SD). Men had higher mean serum myostatin levels versus women (8.3 ± 1.9 vs. 7.2 ± 1.9 ng/mL, *p* = 0.01) and higher serum myostatin levels were associated with higher ALM (*R* = 0.34, *p* = 0.003). Higher serum myostatin levels were associated with lower Matsuda index (*R* = −0.44, *p* = 0.0004), which remained significant after controlling for BMI, VAT, ALM, and sex. In conclusion, higher serum myostatin levels are independently associated with lower insulin sensitivity in adults with overweight/obesity and may be a marker of or play a mechanistic role in the development of insulin resistance.

## 1 INTRODUCTION

The prevalence of type 2 diabetes is increasing in the United States, especially among adults who are overweight/obese (Prevention CfDCa, 2022). Skeletal muscle insulin resistance plays a pathogenic role in the development of type 2 diabetes (Petersen & Shulman, 2002). Skeletal muscle produces myokines, including myostatin, that may play a mechanistic role in the development of insulin resistance. Myostatin, also known as growth differentiation factor‐8 (GDF8), a member of the transforming growth factor‐beta (TGF‐*β*) superfamily of proteins, binds the activin type II A and B (ActRIIA/B) receptors. It negatively regulates skeletal muscle mass by inhibiting both the proliferation and differentiation of myoblasts and the phosphatidylinositol‐3‐kinase (PI3K)/Akt/mammalian target of rapamycin (mTOR) pathway that stimulates protein synthesis (Hittel et al., 2009; Ji et al., 2008). In both preclinical and clinical models, inhibition of myostatin signaling increases muscle mass, and also reduces fat mass and improves insulin sensitivity (Akpan et al., 2009; Garito et al., 2018; Guo et al., 2009; Heymsfield et al., 2021; Lin et al., 2002; Zhao et al., 2005), suggesting that myostatin may have endocrine as well as paracrine effects.

Data regarding the association between serum myostatin levels and insulin sensitivity in humans are discrepant, perhaps in part due to the use of myostatin immunoassays which lack sensitivity and specificity (Bergen et al., 2015) and the inclusion of adults with comorbid conditions or on medications which could have confounded the results. For instance, heart failure (Furihata et al., 2016) and chronic obstructive pulmonary disease (Ju & Chen, 2012) have been associated with higher, while cirrhosis has been associated with lower (Ruiz‐Margáin et al., 2023), serum myostatin levels than controls, and metformin has been reported to induce the expression of myostatin in muscle (Kang et al., 2022). This study addresses these limitations by including only healthy adults with overweight/obesity and measuring serum myostatin levels by liquid chromatography tandem mass spectrometry (LC–MS/MS). In addition, whether there is an association between serum myostatin levels and insulin sensitivity independent of body composition has not been investigated.

We hypothesized that higher serum myostatin levels are associated with lower insulin sensitivity in otherwise healthy adults with overweight/obesity. To address previous limitations in the literature, we excluded adults with type 2 diabetes or other end‐organ dysfunction and quantified serum myostatin levels using LC–MS/MS. We quantified insulin sensitivity using the Matsuda index. We hypothesized that the relationship between myostatin and insulin sensitivity is independent of other risk factors for insulin resistance, such as BMI and visceral adipose tissue (VAT). An a priori decision was made to analyze the data in the entire cohort and control for sex given marked differences in muscle mass between men and women.

## 2 MATERIALS AND METHODS

This study was approved by the Mass General Brigham institutional review board and is compliant with the Health Insurance Portability and Accountability Act (HIPAA). Written informed consent was obtained from all participants prior to any study procedures. All study procedures were performed at the Massachusetts General Hospital Clinical Research Center.

Seventy‐four consecutive participants (34 women, 40 men) with overweight or obesity were recruited over 4 years (2013–2017) through advertisements for an NIH‐funded study investigating the effects of recombinant human growth hormone vs. placebo on bone (ClinicalTrials.gov NCT01724489)—baseline data from this study were analyzed for the current study. A total of 253 participants were screened to achieve a study group of 74 participants. Inclusion criteria included age 18–65 years old and generally healthy with BMI ≥25 kg/m^2^. Exclusion criteria included amenorrhea in premenopausal women; pregnancy or breastfeeding; elevated serum creatinine or thyroid stimulating hormone levels; elevated serum alanine amino transferase levels (>2 times the upper limit of normal); current or prior history of diabetes mellitus, cancer, or other serious chronic disease including of the liver, kidney, heart, or lungs; estrogen, testosterone, growth hormone, glucocorticoid, insulin, or metformin use.

After an overnight fast, participants underwent dual‐energy x‐ray absorptiometry (DXA) and computed tomography (CT) for assessment of body composition. A standard, 75 g, 2‐h oral glucose tolerance test (OGTT) was performed, with blood samples collected at 0, 30, 60, 90, and 120 min. Additional blood was collected for hormone assessment. Exercise was assessed using the Paffenbarger Questionnaire, and hours of vigorous activity (≥6 metabolic equivalents [METs]) a week was recorded.

### 2.1 Dual energy x‐ray absorptiometry

DXA (Hologic Discovery A or Horizon A; Hologic Inc., Bedford, MA, USA) for quantification of appendicular lean mass (ALM) (precision of <2% for lean mass) was performed on all participants. According to 2005 International Society of Clinical Densitometry guidelines, standard cross‐calibration procedures were performed between the Discovery A and Horizon A scanners.

### 2.2 Computed tomography

Single slice CT (LightSpeed Pro, GE Healthcare, Waukesha, WI, USA) of the abdomen through the mid‐portion of the L4 vertebra was performed on all participants. Scan parameters were standardized as follows: 144 mm table height, 80 kV and 70 mA for the abdomen, scanning time of 2 s, 1 cm section thickness, and 48 cm field of view. A threshold set for −50 to −250 Hounsfield units (HU) was used to identify abdominal adipose tissue (Borkan et al., 1982). VAT was separated using manual delineation and reported as cross‐sectional area (CSA) (cm^2^). Osirix software version 3.2.1 was used for analyses (www.osirix‐viewer.com/index.html). Coefficient of variation (CV) for VAT CSA is 2.5%.

### 2.3 Measurement of insulin sensitivity and insulin resistance

The Matsuda index is an index of whole‐body insulin sensitivity derived from a 75 g OGTT. It is strongly correlated with the rate of whole‐body glucose disposal measured by hyperinsulinemic‐euglycemic clamp and has been suggested as the best surrogate for the hyperinsulinemic‐euglycemic clamp (Matsuda & DeFronzo, 1999). The Matsuda index is calculated as follows: 10000/**√**(FG (mg/dL) × FI (μIU/L) × mean glucose × mean insulin) where FG is fasting glucose, FI is fasting insulin, mean glucose is the mean glucose concentration during the 75 g OGTT, and mean insulin is the mean insulin concentration during the 75 g OGTT. The homeostatic model assessment of insulin resistance (HOMA‐IR) is an indirect method for quantifying insulin resistance using fasting glucose and insulin concentrations. The HOMA‐IR is calculated as follows: (FG (mg/dL) × FI (μmol/L))/405. A fasting serum glucose 100–125 mg/dL was characterized as impaired fasting glucose, and a 2‐h serum glucose 140–199 mg/dL during a 75 g OGTT was characterized as impaired glucose tolerance.

### 2.4 Biochemical analysis

Serum samples were run in real time or stored at −80°C. Serum glucose levels were run in real time, and serum insulin levels were run in batch, using commercially available standardized assays. Serum myostatin levels were measured by liquid chromatography tandem‐mass spectrometry (LC–MS/MS) (Brigham Research Assay Core, Boston, MA) with sensitivity of 0.5 ng/mL and intra‐ and inter‐assay CV of 10% and 12%, respectively. Serum myostatin levels were measured in fasting, morning samples given data suggesting that some myokines have a diurnal rhythm (Anastasilakis et al., 2014).

### 2.5 Statistical analysis

Statistical analyses were performed using JMP Statistical Database Software (version 16.2.0; SAS Institute, Cary, NC). Clinical characteristics are reported as mean ± SD. Seven outliers, determined by quantile analysis, were excluded from analyses including the Matsuda index, while five outliers were excluded from analyses using HOMA‐IR. Variables were tested for normality using the Shapiro–Wilk test; non‐normally distributed variables were log‐transformed prior to statistical analysis. Clinical characteristics were compared between men and women using a t‐test or Fisher's exact test. Linear regression analyses were performed to investigate associations between serum myostatin levels and clinical characteristics, body composition, or measures of insulin sensitivity, and Pearson correlation coefficients are reported. Multivariate standard least squares analyses were constructed to investigate the association between serum myostatin levels and measures of insulin sensitivity/resistance independent of body composition, and partial correlation coefficients are reported. Collinearity was assessed using variance inflation factor (VIF); variables with a VIF >5 were excluded from the model. For both the linear regression and multivariate standard least squares analyses, an a priori decision was made to analyze the data in the entire cohort and control for sex given marked differences in muscle mass between men and women. Statistical significance was defined as a two‐tailed *p* < 0.05.

Clinical characteristics, body composition, and HOMA‐IR from subjects in this cohort have been previously published (Dichtel et al., 2022; Haines et al., 2020; Schorr et al., 2016, 2018). However, serum myostatin levels and the Matsuda index have not been reported.

## 3 RESULTS

### 3.1 Clinical characteristics

Mean age was 48 ± 12 years (range 20–65 years) and mean BMI was 33.1 ± 5.6 kg/m^2^ (range 25–50 kg/m^2^) (Table 1). Women had lower mean weight, ALM, ALM/weight, VAT, and serum myostatin levels (*p* < 0.05), and similar mean insulin sensitivity by Matsuda index and insulin resistance by HOMA‐IR, than men (Table 1). However, women had higher serum myostatin levels per kg of ALM than men (Table 1). The presence of impaired fasting glucose and impaired glucose tolerance was similar between women and men (Table 1).

**TABLE 1:** Clinical characteristics (mean ± SD).

	Sexes combined (*n* = 74)	Women (*n* = 34)	Men (*n* = 40)	*p*‐value women vs. men
Clinical characteristics				
White (%)	75.7	76.5	75.0	1.000
Age (years)	48 ± 12	50.7 ± 11.2	45.4 ± 12.8	0.07
Weight (kg)	94.5 ± 16.2	89.5 ± 16.6	98.8 ± 14.7	0.01
Body mass index (BMI) (kg/m^2^)	33.1 ± 5.6	34.2 ± 6.1	32.1 ± 5.0	0.13
Physical activity (h/week)	3.4 ± 4.7	2.1 ± 3.3	4.6 ± 5.5	0.39
Dual‐energy x‐ray absorptiometry				
Appendicular lean mass (ALM) (kg)	25.4 ± 5.3	20.9 ± 3.3	29.2 ± 3.3	<0.0001
ALM/weight	0.27 ± 0.04	0.24 ± 0.03	0.30 ± 0.03	<0.0001
Computed tomography				
Visceral adipose tissue (cm^2^)	155.0 ± 76.9	129.1 ± 48.7	177.1 ± 89.2	0.005
Glucose homeostasis				
Matsuda index	10.5 ± 11.5	12.5 ± 14.0	8.9 ± 8.6	0.07
HOMA‐IR	1.4 ± 1.3	1.1 ± 0.8	1.7 ± 1.5	0.20
Impaired fasting glucose (%)*	13.5	0	20.0	0.10
Impaired glucose tolerance (%)**	21.6	20.6	22.5	1.00
Myokines				
Myostatin (ng/mL)	7.8 ± 1.9	7.2 ± 1.8	8.3 ± 1.9	0.01
Myostatin/ALM (ng/mL/kg)	0.32 ± 0.09	0.35 ± 0.09	0.29 ± 0.07	0.002

* Impaired fasting glucose defined as fasting plasma glucose between 100 and 125 mg/dL.

** Impaired glucose tolerance defined as 2‐h plasma glucose during a 75 g oral glucose tolerance test between 140 and 199 mg/dL.

### 3.2 Associations of serum myostatin levels with clinical characteristics and body composition

Serum myostatin levels were positively associated with ALM and ALM/weight (Table 2), which were not significant after controlling for sex (*p* = 0.10 and *p* = 0.86, respectively). There was no difference in serum myostatin levels between sexes after controlling for ALM (*p* = 0.74). There was a negative linear relationship between serum myostatin levels and age (Table 2), which was not significant after controlling for ALM (*p* = 0.06). There was no association between serum myostatin levels and BMI, weight, hours of vigorous physical activity per week, or VAT (Table 2).

**TABLE 2:** Association of serum myostatin levels with clinical characteristics and body composition.

	Myostatin	
	*R*	*p*
Age	−0.27	0.02
Body mass index	0.00	1.00
Weight	0.20	0.09
Appendicular lean mass (ALM)	0.34	0.003
ALM/weight	0.23	0.049
Physical activity	−0.13	0.33
Visceral adipose tissue	0.05	0.69

### 3.3 Associations of serum myostatin levels, clinical characteristics, and body composition with insulin sensitivity and insulin resistance indices

**FIGURE 1:**
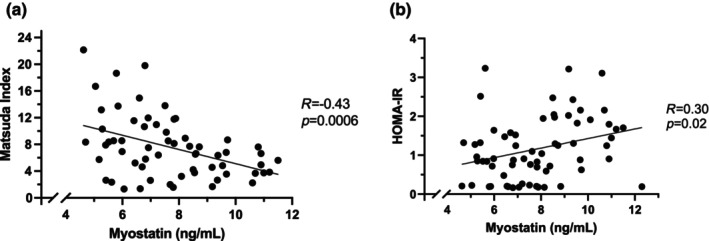
Association between serum myostatin levels and insulin sensitivity by Matsuda index (a) and homeostatic model of insulin resistance (HOMA‐IR) (b).

Higher serum myostatin levels were associated with lower insulin sensitivity by Matsuda index and higher insulin resistance by HOMA‐IR (Figure 1), which remained significant after controlling for sex (*p* = 0.04 and *p* = 0.03, respectively). In a multivariate model including myostatin, BMI, VAT, ALM, and sex, myostatin remained significantly negatively associated with insulin sensitivity by Matsuda index (*p* = 0.04) and insulin resistance by HOMA‐IR (*p* = 0.04).

Higher serum myostatin levels relative to ALM (myostatin/ALM) were not associated with insulin sensitivity by Matsuda index (*R* = −0.11, *p* = 0.41) nor insulin resistance by HOMA‐IR (*R* = 0.22, *p* = 0.07). However, after controlling for BMI, VAT, and sex, myostatin/ALM was negatively associated with insulin sensitivity by Matsuda index (*p* = 0.03), although the association with insulin resistance by HOMA‐IR remained not significant (*p* = 0.07).

Appendicular lean mass was negatively associated with insulin sensitivity by Matsuda index (*R* = −0.35, *p* = 0.006), which was not significant after adjusting for weight (*p* = 0.55); ALM was not associated with insulin resistance by HOMA‐IR (*R* = 0.05, *p* = 0.68). Similarly, ALM/weight was not associated with Matsuda index (*R* = 0.003, *p* = 0.98) nor HOMA‐IR (*R* = −0.13, *p* = 0.29).

## 4 DISCUSSION

Our data suggest that higher serum myostatin levels are associated with lower insulin sensitivity in otherwise healthy adults, aged 20–65 years, with overweight/obesity, independent of other risk factors known to be associated with insulin resistance, namely BMI, visceral adiposity, and sex. Previous studies investigating the association between serum myostatin levels and insulin sensitivity have been inconsistent, perhaps because they included adults with comorbid conditions or on medications which could have confounded results, used myostatin immunoassays that lacked sensitivity or specificity, and did not control for other factors associated with insulin resistance. We addressed these limitations by studying a group of otherwise healthy adults with overweight/obesity and without type 2 diabetes or other end‐organ dysfunction, and by measuring fasting serum myostatin levels using a validated LC–MS/MS assay with excellent sensitivity and specificity.

Myostatin inhibition in preclinical and clinical models increases muscle mass, reduces fat mass, and improves insulin sensitivity (Akpan et al., 2009; Garito et al., 2018; Guo et al., 2009; Heymsfield et al., 2021; Lin et al., 2002; Zhang et al., 2012, 2005), which may be mediated in part by myostatin's effects on body composition (Akpan et al., 2009; Guo et al., 2009; Lin et al., 2002; Zhao et al., 2005). However, the improvement in insulin sensitivity with inhibition of myostatin signaling has been demonstrated to be independent of increases in muscle mass or decreases in fat mass (Garito et al., 2018; Guo et al., 2009), which suggests that myostatin may have unfavorable effects on insulin sensitivity independent of body composition. However, an association between serum myostatin levels and insulin sensitivity independent of body composition and other risk factors for insulin resistance, namely higher BMI (Miao et al., 2020), higher VAT (Liu et al., 2010), and male sex (Tramunt et al., 2020), has not been reported using a validated myostatin LC–MS/MS assay. Previous studies that investigated the association between serum myostatin levels and insulin sensitivity or resistance indices in adults with obesity and without type 2 diabetes were discrepant (Amor et al., 2019; Brandt et al., 2012; Carvalho et al., 2018; Hittel et al., 2010; Kern‐Matschilles et al., 2022; Toloza et al., 2018). Studies that did not find an association between plasma myostatin levels and insulin sensitivity by hyperinsulinemic‐euglycemic clamp or insulin resistance by HOMA‐IR or HOMA2‐IR among adults of a similar mean age and BMI did not exclude adults with end‐organ dysfunction and measured myostatin levels by immunoassay (Toloza et al., 2018) or did not exclude adults on metformin and did not measure myostatin levels fasting (Brandt et al., 2012). Our results are consistent with two studies in which fasting serum myostatin levels measured by immunoassay were negatively correlated with insulin sensitivity by Matsuda index or quantitative insulin‐sensitivity check index (QUICKI) and positively correlated with HOMA‐IR among adults across a wide BMI range and similar age range to our study without significant cardiovascular or pulmonary disease or metformin use (Amor et al., 2019; Carvalho et al., 2018). We add to the literature by measuring fasting serum myostatin levels by LC–MS/MS, and demonstrating that the associations are independent of BMI, VAT, ALM, and sex, in otherwise healthy adults with overweight/obesity.

Although this study and prior studies have reported associations with serum myostatin levels and sex, age, and body composition, we demonstrate that higher serum myostatin levels are associated with lower insulin sensitivity independent of body composition, sex, and age. A number of studies agree with our findings that fasting serum myostatin levels are higher in men versus women (Bergen et al., 2015; Garcia‐Fontana et al., 2016; Kurose et al., 2021; Toloza et al., 2018) and decline with aging among adults without type 2 diabetes (Bergen et al., 2015; Lakshman et al., 2009). Discrepant results in other studies may have been because blood samples were not collected fasting, myostatin levels were measured by immunoassay, and/or the studies did not exclude adults with end‐organ dysfunction (Brandt et al., 2012; Lakshman et al., 2009; Ratkevicius et al., 2011). We did find that sex and age differences in serum myostatin levels were not significant after controlling for sex‐ and age‐related differences in ALM, which would align more with two cross‐sectional studies that measured fasting serum myostatin levels by LC–MS/MS and reported that serum myostatin levels were not associated with age (Peng et al., 2022; Semba et al., 2019). Prior studies have reported that myostatin levels are positively associated with muscle mass (e.g., lean mass) and relative muscle mass (e.g., lean mass/weight) (Bergen et al., 2015; Carvalho et al., 2018; Kurose et al., 2021; Toloza et al., 2018), which agree with our results. Myostatin levels may increase with increasing muscle mass in order to act as a negative regulator of muscle growth as myostatin is a known inhibitor of myocyte differentiation and proliferation (Hittel et al., 2009; Ji et al., 2008). Alternatively, since lean mass in adults with obesity is characterized by greater myosteatosis (Miljkovic & Zmuda, 2010) and skeletal muscle extracellular matrix (Sachs et al., 2019), myostatin may mediate the relationship between greater myosteatosis and/or skeletal muscle extracellular matrix and lower insulin sensitivity in adults with obesity. This possibility requires further investigation.

Adults with overweight/obesity generally have higher muscle mass concurrent with their higher weight, with weight being a potential confounder in the relationship between ALM and Matsuda index. Consistent with this, ALM and weight are negatively associated with insulin sensitivity by Matsuda index, and the association between ALM and Matsuda index is no longer significant after controlling for weight. In contrast, greater ALM is associated with both higher serum myostatin levels and lower insulin sensitivity by Matsuda index. However, ALM is not a cofounder in the relationship between myostatin and Matsuda index because the negative association between serum myostatin levels and insulin sensitivity by the Matsuda index remains significant after controlling for ALM.

Study strengths include the use of a sensitive and specific myostatin LC–MS/MS assay that can distinguish between the latent and mature forms of myostatin, only the latter of which is measured. Myostatin immunoassays can both overestimate and underestimate serum myostatin concentrations due to cross‐reactivity with GDF11 (Rodgers & Eldridge, 2015) and the presence of myostatin‐protein complexes that block epitopes recognized by myostatin antibodies (Ratkevicius et al., 2011). Myostatin immunoassays are also limited by relatively low sensitivity, which is relevant as myostatin is present in relatively low concentrations in serum. Previous literature has shown that there is no significant correlation between myostatin levels measured by ELISA versus LC–MS/MS (Semba et al., 2019). One limitation of myostatin LC–MS/MS assays is that measuring serum levels of mature myostatin may not reflect myostatin bioavailability and activity due to the binding of mature myostatin to plasma binding proteins, such as follistatin (Peng et al., 2022). Although another limitation is that circulating levels of myostatin do not necessarily reflect tissue myostatin expression, previous studies have shown excellent correlation between myostatin tissue expression and secretion (Brandt et al., 2012; Hittel et al., 2009), and studies have shown that greater skeletal muscle myostatin mRNA and protein expression are associated with insulin resistance (Hittel et al., 2009; Park et al., 2006; Ryan et al., 2013). The cross‐sectional nature of this study is a limitation, such that the association between myostatin and insulin sensitivity does not prove causality or the directionality of effect. In addition, a single fasting myostatin sample may not reflect 24‐h serum levels of myostatin, whose secretion is at least in part regulated by exercise. Interventional studies in which the myostatin signaling pathway is manipulated are needed to elucidate the mechanism of action of myostatin on insulin sensitivity. Although we did not perform hyperinsulinemic‐euglycemic clamps to directly measure insulin sensitivity, the Matsuda index is highly correlated with the rate of whole‐body glucose disposal during the hyperinsulinemic‐euglycemic clamp (Matsuda & DeFronzo, 1999). We were underpowered to detect an interaction by sex in this study.

In conclusion, our data suggest that higher serum myostatin levels are associated with lower insulin sensitivity in otherwise healthy adults, age 20–65 years, with overweight/obesity, independent of other risk factors for insulin resistance. We add to the literature by investigating associations between serum myostatin levels and sex, age, lean mass, and insulin sensitivity using a sensitive and specific myostatin LC–MS/MS assay. These data suggest that serum myostatin levels may be a marker of or play a mechanistic role in the development of insulin resistance. Future prospective, longitudinal studies should investigate myostatin as a biomarker or potential therapeutic target for insulin resistance.

## AUTHOR CONTRIBUTIONS

LED was responsible for investigation, formal analysis, writing review and editing, and funding acquisition. AK was responsible for investigation and writing review and editing. BB was responsible for investigation, data curation, and writing review and editing. GS was responsible for data curation, visualization, and writing review and editing. AVG was responsible for investigation and writing review and editing. MAB was responsible for software, investigation, resources, writing review and editing, supervision, project administration and funding acquisition. MSH was responsible for conceptualization, methodology, validation, formal analysis, investigation, resources, data curation, writing of the original draft and review and editing, visualization, supervision, project administration, and funding acquisition.

## FUNDING INFORMATION

Funding support was provided by the following: NIH K23DK113220 (Dichtel); NIH K23DK115903 (Haines); Boston Area Diabetes Endocrinology Research Center (Haines); NIH K24DK109940 (Bredella); NIH R01DK095792; NIH T32DK007028; NIH 1UL1TR001102; 8UL1TR000170, Harvard Clinical and Translational Science Center, from the National Center for Advancing Translational Science; UL1RR025758, Harvard Clinical and Translational Science Center, from the National Center for Research Resources; M01‐RR‐01066, from the National Center for Research Resources.

## CONFLICT OF INTEREST STATEMENT

LED has received study medication from Pfizer, research support from Perspectum Ltd. and research support from Lumos Pharma, all per investigator‐initiated request. She has additional research support from Recordati and Novo Nordisk. She has equity in Marea Therapeutics and Merida Biosciences. She is a consultant for Lumos Pharma, Novo Nordisk and Flare Therapeutics. She is a fellow at Third Rock Ventures through the Mass General Brigham's Innovation Fellows Program but remains a full‐time employee of Mass General Brigham during the course of this educational program (10/1/2022–9/30/2024). Dr. Dichtel's financial interests were reviewed and are managed by Massachusetts General Hospital and Mass General Brigham in accordance with their conflict‐of‐interest policies. MSH, AK, BB, GS, AVG, and MAB have nothing to disclose.

## ETHICS STATEMENT

This study was approved by the Mass General Brigham institutional review board (IRB) and is compliant with the Health Insurance Portability and Accountability Act (HIPAA). Written informed consent was obtained from all participants prior to any study procedures. All procedures were conducted following the IRB‐approved protocol.

## Data Availability

The datasets generated during and/or analyzed during the current study are not publicly available but are available from the corresponding author on reasonable request.
